# Opposing Roles for the α Isoform of the Catalytic Subunit of Protein Phosphatase 1 in Inside–Out and Outside–In Integrin Signaling in Murine Platelets

**DOI:** 10.3390/cells12202424

**Published:** 2023-10-10

**Authors:** Tanvir Khatlani, Subhashree Pradhan, Kimberly Langlois, Deepika Subramanyam, Rolando E. Rumbaut, K. Vinod Vijayan

**Affiliations:** 1 Cardiovascular Research Section, Department of Medicine, Baylor College of Medicine, Houston, TX 77030, USA; 2 Center for Translational Research on Inflammatory Diseases (CTRID), Michael E. DeBakey Veterans Affairs Medical Center (MEDVAMC), Houston, TX 77030, USA; 3 Pulmonary Section, Department of Medicine, Baylor College of Medicine, Houston, TX 77030, USA

**Keywords:** platelets, protein phosphatase 1 alpha, p38 mitogen-activated protein kinase, fibrinogen

## Abstract

Platelet activation during hemostasis and thrombosis is facilitated by agonist-induced inside–out and integrin α_IIb_β_3_-initiated outside–in signaling via protein kinases and phosphatases. Pharmacological inhibitor studies suggest that the serine/threonine protein phosphatase 1 (PP1) promotes platelet activation. However, since phosphatase inhibitors block all the isoforms of the catalytic subunit of PP1 (PP1c), the role of specific PP1c isoform in platelet signaling remains unclear. Here, we employed a platelet-specific PP1cα^−/−^ mice to explore the contribution of a major PP1 isoform in platelet functions. Loss of PP1cα moderately decreased activation of integrin α_IIb_β_3_, binding of soluble fibrinogen, and aggregation to low-dose thrombin, ADP, and collagen. In contrast, PP1cα^−/−^ platelets displayed increased adhesion to immobilized fibrinogen, fibrin clot retraction, and thrombus formation on immobilized collagen. Mechanistically, post-fibrinogen engagement potentiated p38 mitogen-activated protein kinase (MAPK) activation in PP1cα^−/−^ platelets and the p38 inhibitor blocked the increased integrin-mediated outside–in signaling function. Tail bleeding time and light-dye injury-induced microvascular thrombosis in the cremaster venules and arterioles were not altered in PP1cα^−/−^ mice. Thus, PP1cα displays pleiotropic signaling in platelets as it amplifies agonist-induced signaling and attenuates integrin-mediated signaling with no impact on hemostasis and thrombosis.

## 1. Introduction

At the site of vascular injury or ruptured atherosclerotic plaques, exposed extracellular matrix proteins such as collagen and the generated agonist such as thrombin, thromboxane and adenosine diphosphate (ADP) engage with their respective platelet receptors to initiate agonist-induced inside–out signaling [1]. This signaling triggers major platelet integrin α_IIb_β_3_ to bind soluble fibrinogen and support platelet aggregation. Fibrinogen binding to integrin α_IIb_β_3_ generates a second set of signals referred to as outside–in signaling, which controls the platelet cytoskeletal remodeling and functions such as adhesion and fibrin clot retraction [2,3]. Both inside–out and outside–in signaling contribute to platelet activation and its ability to form stable platelet thrombi.

Protein kinases have held the center stage in our understanding of the agonist and integrin-mediated signaling pathways. As models of signal transduction have been refined to include protein phosphorylation and dephosphorylation, the contribution of the protein phosphatases to platelet signaling and function has garnered fresh attention [4]. Indeed, in quantitative phospho-proteome studies that examined basal, and ADP-stimulated platelets, temporal phosphorylation and dephosphorylation patterns, predominantly in the serine and threonine residues of proteins that engaged the signaling networks governing platelet function were observed [5,6].

Protein phosphatase 1 holoenzyme is among the major serine/threonine phosphatases in eukaryotes and contains a catalytic (PP1c) and regulatory subunit (PP1r) [7]. PP1c has three isoforms PP1cα, PP1cβ, and PP1cγ, all of which are expressed in platelets with the copy number of PP1cα being the highest [8]. Historically, the role of PP1 in platelet biology has been examined using pharmacological agents such as calyculin A and okadaic acid. These agents impaired human platelet functions [9] including aggregation [10,11], secretion [12], the adhesion/spreading on immobilized fibrinogen [13], and clot retraction [14,15] suggesting that PP1 promotes agonist-induced inside–out and integrin-initiated outside–in signaling in platelets. However, since these inhibitors cannot discriminate the different isoforms of PP1c, pharmacological agents are inadequate to inform the isoform-specific role of PP1c in platelet signaling. We have previously generated a platelet-specific PP1cα null mice model and observed decreased thrombin-induced inside–out platelet signaling [16]. Using this genetic model, we explored the impact of PP1cα on inside–out and outside signaling functions in platelets. We report that PP1cα positively regulates agonist-induced inside–out signaling responses such as integrin α_IIb_β_3_ activation, soluble fibrinogen binding, and aggregation in response to low doses of agonist. Concurrently, PP1cα negatively regulates integrin outside–in signaling responses such as platelet adhesion and fibrin clot retraction. Consistent with an opposing role for PP1cα in agonist-induced inside–out and integrin-induced outside signaling, loss of PP1cα did not impact in vivo hemostasis and thrombosis.

## 2. Materials and Methods

### 2.1. Materials

The reagents used in this study were mostly obtained from Sigma-Aldrich (St. Louis, MO, USA). Anti-CD41(α_IIb_) and anti-CD62 (P-selectin) antibodies tagged to fluorescein isothiocyanate (FITC) were obtained from BD Bioscience (San Jose, CA, USA). Phycoerythrin-tagged antibodies that recognize the active form of murine α_IIb_β_3_ (JON/A) were purchased from Emfret Analytics (Eibelstadt, Germany). Thrombin was brought from Hematologic Technologies Inc. (Essex Junction, VT, USA). ADP and collagen were purchased from Helena Laboratories (Beaumont, TX, USA). Thromboxane analog (U44619) and water-soluble p38 SB203580 hydrochloride were ordered from Bio-Techne Corporation (Minneapolis, MN, USA). Fibrinogen and Alexa 488-tagged fibrinogen were purchased from Enzyme Research Laboratories Inc. (South Bend, IN, USA) and Invitrogen, (Carlsbad, CA, USA), respectively. Anti-phospho-p38 and p38 antibodies were obtained from Cell Signaling (Boston, MA, USA).

### 2.2. Mice

Approval from Baylor College of Medicine IACUC (Institutional Animal Care and Use Committee) was sought prior to initiating all animal studies. Mice lacking PP1cα conditionally in platelets were generated as we described previously [16]. Briefly, *ppp1ca*-*loxP* flox (*fl*) mice [17] were mated with megakaryocyte/platelet-specific *Pf4 Cre* knock-in mice (Jackson Laboratory) [18] to generate *PP1c*α knock-out mice. For most studies, 8–16-week-old male and female littermates (*PP1c*α *fl*/fl and *Cre* positive, referred to as PP1cα^−/−^) and *PP1c*α *fl*/fl and *Cre* negative, referred to as wild type (WT) were employed. The only exception is the use of male mice in intravital microscopy studies on cremaster muscles.

### 2.3. Mice Platelet Preparation

Mice were anesthetized with isoflurane and blood was collected into the anticoagulant acid-citric acid-dextrose (ACD) in a 1:10 (*v*/*v*) ratio from the inferior vena cava, as we described [19]. Collected blood was then diluted in a 1:1 ratio with Dulbecco’s phosphate buffered saline (D-PBS) which contains one part of ACD to nine parts of D-PBS. The first centrifugation at 68× *g* for 10 min provided platelet-rich plasma (PRP). The second centrifugation of PRP at 754× *g* for 10 min yielded a platelet pellet that was subsequently washed with D-PBS. Platelets were then gently resuspended in D-PBS containing 0.005 U/mL apyrase. Coulter counter (Beckman–Coulter (Z1), Miami, FL, USA) was used to count platelets, which were adjusted to the final concentration of 2.5 × 10^8^/mL. All studies with washed platelets were initiated after resting for one hour.

### 2.4. Flow Cytometry Studies for Integrin Activation, Binding of Soluble Fibrinogen, Granule Secretion, and Platelet Aggregation

For these studies, platelets were further diluted to 2.5 × 10^7^/mL with Tyrode’s buffer containing CaCl_2_ (1.8 mM) and MgCl_2_ (0.49 mM). Agonist including thrombin (0.02 U/mL, 0.05 U/mL), ADP (2.5 μM, 10 μM), collagen (0.5, 0.75, 1, 1.5 and 2.5 μg/mL) and U46619 (0.25, 0.50 μM) was used to stimulate platelets. Depending on the assays to be studied, we employed JON/A tagged PE antibody, Alexa 488-tagged soluble fibrinogen, FITC conjugated CD62P antibody and CD41 tagged PE antibody and analyzed platelets in a flow cytometer (EPICS-XL, Beckman Coulter, Miami, FL, USA). FITC or PE-tagged isotype antibodies served as control. To study agonist-induced release of ATP, luciferin/luciferase reagent was added to agonist-challenged washed platelet and evaluated in a lumi-aggregometer (Chrono-Log Corp. Havertown, PA, USA) [20]. To study aggregation, washed platelets (225 μL of 2.5 × 10^8^/mL) were stimulated with different concentrations of agonist under stirring conditions (1200 rpm) in an eight-channel Bio/Data PAP-8 aggregometer (Biodata Corporation, Horsham, PA, USA). Final aggregation after 10 min of agonist stimulation was recorded.

### 2.5. p38 MAPK Immunoblotting Studies

Platelets were left suspended on BSA (5 mg/mL) substrate for 30 min or allowed to adhere to immobilized fibrinogen (100 ug/mL) for 15 and 30 min. Following washing, platelet lysate was separated on 10–12% SDS-PAGE (sodium dodecyl sulfate-polyacrylamide gel electrophoresis), transferred to nitrocellulose membrane, and then immunoblotted with antibodies to phospho-Thr180, Tyr182 (phospho p38; surrogate marker of p38 activation) and p38 MAPK. HRP-conjugated secondary antibodies were used later, and the membrane was developed using chemiluminescence (ECL). Densitometry of p38 signals was performed using Image J (1.53k/Java/8.0_172 964 bits).

### 2.6. Platelet Adhesion, Clot Retraction and In Vitro Thrombus Formation

For static adhesion assays, ninety-six well plates were covered with fibrinogen (100 μg/mL) and subsequently blocked with 5 mg/mL of Bovine Serum Albumin (BSA). Wells coated with BSA served as controls. Untreated and water-soluble p38 inhibitor (SB203580 10 μM) treated WT and PP1cα^−/−^ washed platelets (1 × 10^7^) were incubated for 60 min. Following washing, the acid phosphatase activity of bound platelets was used to quantify platelet adhesion. A standard curve of acid phosphatase 405 absorbance with varying platelet counts was used to obtain the adhered platelet number. Percent platelet adhesion as obtained we have previously reported [19,20]. For clot retraction assays, platelet-rich mouse plasma (500 μL of 2.5 × 10^8^ platelets/mL) containing 3 mM CaCl_2_ was left untreated or treated with SB203580 (10 μM) and then challenged with 1 U/mL thrombin for 60 min. The volume of liquid that was not incorporated into the clot was measured. To ascertain the volume of the clot, we subtracted the measured volume from the initial 500 μL volume and expressed it as a percentage of the starting volume. For in vitro platelet adhesion and thrombus under flow conditions, whole blood collected in PPACK dihydrochloride from wild type (WT) and PP1cα^−/−^ mice were perfused over collagen type 1 at 1000^s−1^ using a Bio Flux 1000Z Flexion system (Flexion Biosciences, Oakland, CA, USA). Following 4 min of perfusion, epifluorescence microscopy was used to collect multiple images of platelet adhesion and thrombus formation at different sites by an investigator who was blinded to the genotype. Platelet adhesion and thrombus formation were reported as integrated fluorescence intensity using the Bio flux Montage software (Version 7.8.4.0).

### 2.7. Intra Vital Microscopy and Vivo Platelet Thrombus Formation

To study microvascular thrombosis in the venules and arterioles of the cremaster muscle, we used a light/dye–induced endothelial injury model and evaluated it by intravital microscopy as we previously studied [21,22]. Briefly, following a 50 mg/kg phenobarbital sodium-induced anesthesia, male mice were subjected to tracheotomy and cannulation of the internal jugular vein and common carotid arteries. While the former procedure assisted in breathing, the latter enabled the delivery of agents and continuous monitoring of heart rate and blood pressure. The cremaster microvascular bed of the mice was then exposed and equilibrated for at least 30 min with a saline solution buffered with bicarbonate (pH 7.35–7.45) at 35 °C. Through the jugular vein, a 5% FITC-labeled dextran (10 mL/Kg) was injected. Venules and arterioles for the study were then selected and the diameter and velocity of blood flow through these vessels were monitored with a Doppler velocimeter (Microcirculation Research Institute, College Station, TX, USA). 100 μm of the vessels were then exposed to filtered excitation light at 0.6 W/cm^2^ from a 175 W xenon lamp to trigger a photochemical injury that triggers thrombosis. After continuously applying epi-illumination, the time of onset for the initiation of platelet aggregates (thrombus onset) and the time required to occlude the vessel and stop the flow for at least 60 s were monitored. For each mouse, thrombosis was triggered in one or two venules and arterioles, and the average of the results reported. The investigators performing these studies were not aware of the genotype of the mice.

### 2.8. Hemostasis Studies

WT and PP1cα^−/−^ mice were anesthetized and 1 mm of the tip of the tail was clipped with a sterile blade and the time required to stop the bleeding was recorded, as we described before [19,20]. After the clip, the bleeding tail was submerged in a PBS solution for 30 s and then subsequently transferred to a new PBS-containing tube until there was no evidence of blood in the PBS solution. The time from the cut to the termination of blood flow was recorded as tail bleeding time.

### 2.9. Statistics

Results are shown as mean +/− standard deviation. In studies that analyzed two experimental groups, a Students’ paired t-test was considered. For the analysis of three or more experimental groups, one-way ANOVA and Tukey multiple comparisons were employed. Analysis was conducted using GraphPad Prism 9 (GraphPad Software, San Diego, CA, USA) and studies were considered significant only if the *p*-value was <0.05.

## 3. Results

### 3.1. Loss of PP1cα Moderately Reduced Low Dose Agonist-Induced Integrin Signaling

We previously generated a conditional platelet PP1cα^−/−^ mice and observed reduced platelet aggregation and soluble fibrinogen binding to a lower dose of thrombin (0.02 U/mL). At higher thrombin concentration (1 U/mL), aggregation and soluble fibrinogen binding were similar between wild-type (WT) and PP1cα^−/−^ platelets [16]. Like PP1cα^−/−^ platelets, PP1cγ^−/−^ platelets also demonstrated moderately decreased aggregation selectively to low but not high doses of thrombin [19]. Due to the potential compensatory effects from other PP1c isoforms during stimulation with high agonist concentrations, we performed most of our studies in this report with a low dose of agonist.

**Figure 1:**
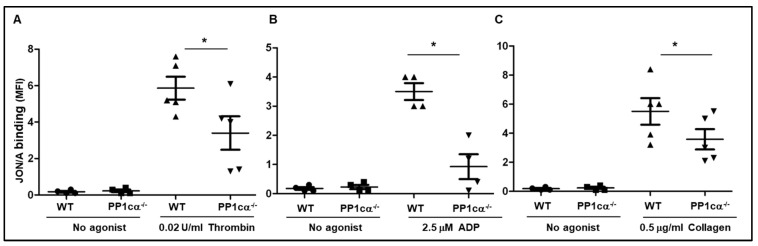
Loss of PP1cα moderately decreased agonist-induced integrin α_IIb_β_3_ activation. Washed platelets from wild type (WT) and PP1cα^−/−^ mice under basal (no agonist) or challenged with low-dose agonists such as thrombin (**A**), ADP (**B**), and collagen (**C**) were incubated with anti-JON/A-PE (recognizes active murine α_IIb_β_3_) and subjected to flow cytometry. *n* = 4–5. * *p* < 0.05.

Since activation of integrin α_IIb_β_3_ is a consequence of inside–out signaling triggered by an agonist, we examined the activation status of murine integrin α_IIb_β_3_ using JON/A antibody. Compared to the WT, PP1cα^−/−^ platelets bound significantly less JON/A antibody following thrombin (0.02 U/mL) stimulation (Figure 1A). Extending these studies to low doses of other agonists, we observed moderately decreased JON/A binding to PP1cα^−/−^ platelets in response to ADP (2.5 μM) (Figure 1B) and collagen (0.5 μg/mL) (Figure 1C). 

**Figure 2:**
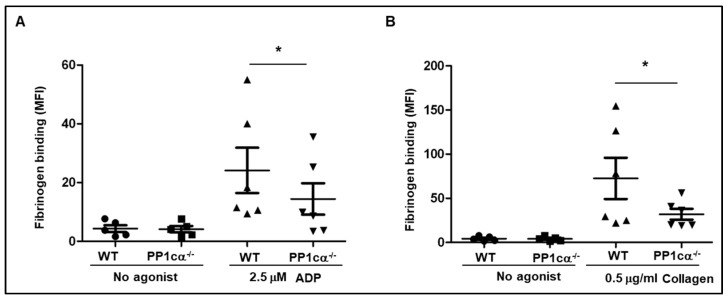
Soluble fibrinogen binding was moderately decreased in PP1cα^−/−^ platelets. Washed platelets. from wild type (WT) and PP1cα^−/−^ mice were stimulated with low dose agonist (**A**) ADP, (**B**) Collagen or left unstimulated (no agonist), and the binding of fluorescent Alexa 488 fibrinogen measured using flow cytometry as mean fluorescence intensity (MFI). *n* = 6. * *p* < 0.05.

We then analyzed the binding of soluble fibrinogen to WT and PP1cα^−/−^ platelets. Compared to the WT platelets, PP1cα^−/−^ platelets revealed a moderate but significant decrease in soluble fibrinogen binding to ADP (2.5 μM) (Figure 2A) and collagen (0.5 μg/mL) (Figure 2B). We had previously reported a decreased soluble fibrinogen-binding response to 0.02 U/mL thrombin by PP1cα^−/−^ platelets [16]. 

**Figure 3:**
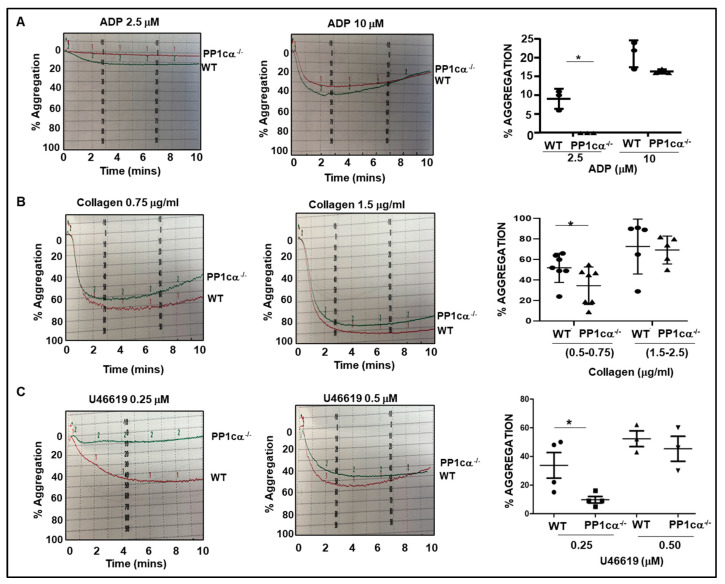
Moderately reduced aggregation of PP1cα^−/−^ platelets to low but not to high dose agonists. Washed platelets were subjected to a range of agonist concentrations (**A**) ADP, (**B**) Collagen, (**C**) thromboxane analog U46619, and final percent platelet aggregation was recorded. *n* = 3–7. * *p* < 0.05.

Because soluble fibrinogen binding facilitates platelet aggregation, we examined agonist-induced aggregatory response. Aggregation of platelets to ADP (2.5 μM) (Figure 3A). collagen (0.5–0.75 μg/mL) (Figure 3B) and thromboxane analog; U46619 (0.25 mM) (Figure 3C) was moderately decreased in PP1cα^−/−^ platelets. Similarly, we previously reported reduced aggregation to 0.02 U/mL thrombin in PP1cα^−/−^ platelets [16]. Aggregation was comparable in WT and PP1cα^−/−^ platelets at higher agonist concentrations (Figure 3) Taken together, our studies indicate that loss of PP1cα modestly decreased activation of integrin α_IIb_β_3_, binding of soluble fibrinogen, and aggregation of platelets. Moreover, the data suggests that PP1cα positively regulates the low-dose agonist-induced inside–out signaling process.

### 3.2. Loss of PP1cα Enhanced Activation of p38 MAPK and Integrin α_IIb_β_3_ Outside–In Signaling

**Figure 4:**
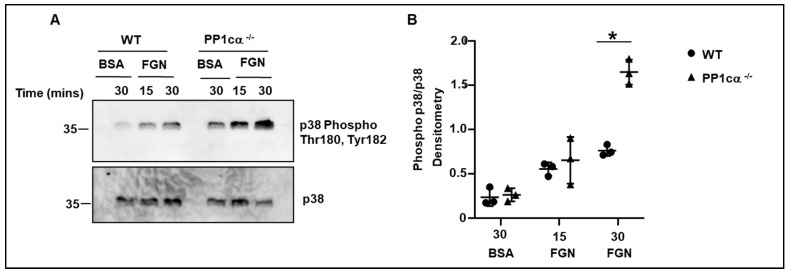
Increased activation of mitogen-activated protein kinase (MAPK) p38 in fibrinogen-adhered PP1cα^−/−^ platelets. (**A**) Washed platelets were maintained in suspension over bovine serum albumin (BSA) or allowed to adhere to fibrinogen (FGN). Lysate was immunoblotted with anti-phospho Thr180, Tyr182 p38, and anti-p38 antibodies. (**B**) Densitometry quantification of three independent experiments. * *p* < 0.05.

**Figure 5:**
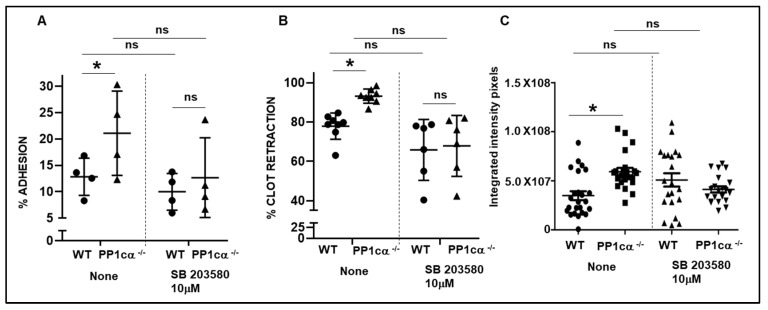
PP1cα^−/−^ platelets showed increased integrin outside–in signaling functions and the p38 inhibitor blocked these functions. (**A**) Increased adhesion of PP1cα^−/−^ platelets to fibrinogen at 60 min was blocked by water-soluble p38 inhibitor SB203580. *n* = 4. (**B**) Enhanced fibrin clot retraction from platelet-rich plasma of PP1cα^−/−^ mice was attenuated with a p38 inhibitor. *n* = 6–8. (**C**) Whole blood from PP1cα^−/−^ mice perfused over immobilized collagen at 1000^s−1^ resulted in an increased in vitro thrombus coverage that was blocked by a p38 inhibitor. Images acquired after 4 min of perfusion were quantified as integrated fluorescence intensity from three independent experiments. * *p* < 0.05. The differences between p38 inhibitor-treated platelets and untreated platelets were not statistically significant (*p* > 0.05; ns).

We previously demonstrated robust activation of p38 mitogen-activated protein kinase (MAPK) in PP1cα depleted heterologous α_IIb_β_3_ overexpressing 293 cells. Importantly, this effect was specific to PP1cα but not to other isoforms, and to p38 MAPK but not to other MAPKs [23]. Therefore, in this study, we evaluated the phosphorylation of residues Thr^180^ and Tyr^182^ of p38 in WT and PP1cα^−/−^ platelets. Phosphorylation of Thr^180^ and Tyr^182^ of p38 was induced following adhesion to immobilized fibrinogen in both WT and PP1cα^−/−^ platelets. Importantly, the adhesion of PP1cα^−/−^ platelets led to a robust activation of p38 at 30 min (Figure 4A,B). Thus, loss of PP1cα potentiates p38 MAPK activation in fibrinogen-adhered platelets. Following integrin-fibrinogen binding there is a reorganization of the platelet cytoskeleton that impacts platelet functions such as adhesion and clot retraction. Therefore, we studied these functions in WT and PP1cα^−/−^ platelets. Significantly increased adhesion to immobilized fibrinogen was observed with PP1cα^−/−^ platelets when compared to the WT platelets (Figure 5A). p38 inhibitor moderately reduced adhesion in both WT and PP1cα^−/−^ platelets. More importantly, p38 inhibitor SB 203,580 blocked the increased adhesiveness of PP1cα^−/−^ platelets on immobilized fibrinogen (Figure 5A). We also studied another function dependent on outside–in signaling, namely fibrin clot retraction. Compared to the platelet-rich plasma (PRP) from WT mice, PP1cα^−/−^ mice displayed significantly increased clot retraction. The addition of the p38 inhibitor moderately reduced clot reaction in both WT and PP1cα^−/−^ platelets. Importantly, the p38 inhibitor ablated the increased clot retraction by PP1cα^−/−^ platelets (Figure 5B). Since exposed collagen can support thrombus growth under shear stress, we perfused blood from both mice on immobilized collagen at 1000^-s^ and studied thrombus formation. Significantly increased platelet adhesion and thrombus formation on immobilized collagen shown by PP1cα^−/−^ mice was blocked by p38 inhibitor (Figure 5C). Collectively, our studies indicate that the loss of PP1cα in platelets leads to moderately enhanced outside–in α_IIb_β_3_ signaling in part via p38 MAPK.

### 3.3. Loss of PP1cα Does Not Impact Secretion and In Vivo Hemostasis and Thrombosis

**Figure 6:**
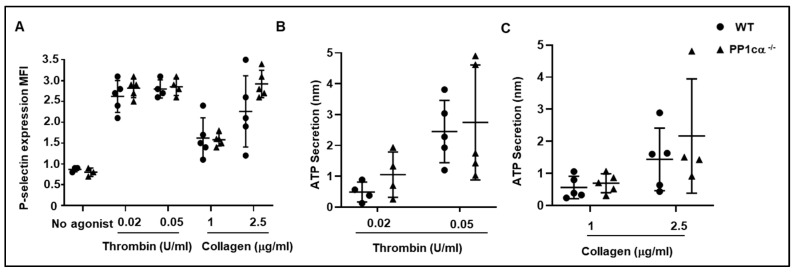
Platelet granule secretion is not affected by the loss of PP1cα. (**A**) Wildtype (WT) (●) and PP1cα^−/−^ platelets (▲) (resting or agonist activated) were challenged with agonists and secretion of a granule was studied using fluorescent anti-P-selectin antibody via flow cytometry as mean fluorescence intensity (MFI). *n* = 5. The release of dense granule content ATP is evaluated in washed platelets in response to (**B**) thrombin and (**C**) collagen by studying luciferin/luciferase. *n* = 4.

**Figure 7:**
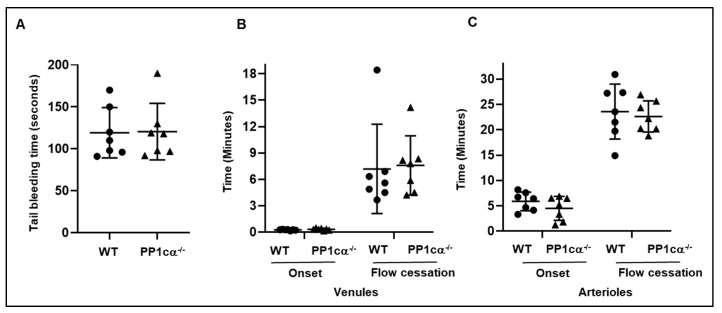
In vivo hemostasis and thrombosis are not affected by the loss of PP1cα. (**A**) Time to achieve hemostasis after a tail bleeding assay from six wild-type (WT) and PP1cα^−/−^ mice were recorded. Light-dye-induced microvascular thrombosis was studied in venules (**B**) and arterioles (**C**) of the cremaster muscle from WT and PP1cα^−/−^ mice using intraviral microscopy. The onset of thrombosis (onset) and cessation of blood flow following injury were studied.

Alpha and dense granule secretion is one of the functional consequences of platelet activation and therefore we assessed P-selectin expression and ATP release following agonist stimulation. P-selectin and ATP secretion were comparable between WT and PP1cα^−/−^ platelets exposed to low and high concentrations of thrombin and collagen (Figure 6A–C). To study if altered in vitro signaling responses displayed by PP1cα^−/−^ platelets impact in vivo hemostasis, we performed mouse tail clip assays. WT and PP1cα^−/−^ mice had similar tail bleeding times (Figure 7A). To evaluate if loss of PP1cα impacts thrombosis, we induced microvascular thrombosis in WT and PP1cα^−/−^ mice using a light/dye-induced injury model. The time to initiate a thrombus (onset time) and the time to occlude the vessel (occlusion time) in the venules and arterioles of the PP1cα^−/−^ mice were similar to that of WT mice (Figure 7B,C). Thus, loss of PP1cα in platelets did not impact in vivo hemostasis and thrombosis.

## 4. Discussion

PP1 positively regulates platelet functions based on previous studies using Ser/Thr phosphatase inhibitors [9,10,11,12,13,14]. Although such chemical inhibitors represent a useful tool, they lack specificity to discriminate protein phosphatase 1, protein phosphatase 2A, and protein phosphatase 4 and their respective isoforms [24]. Thus, it remains possible that the observed phenotype in pharmacological studies may be due to the combined inhibitory effect of multiple phosphatases. Here using a genetic approach, our studies uncovered a subtle but intriguing phenotype for PP1cα in platelet signaling. Specifically, PP1cα was shown to amplify low-dose agonist-induced inside–out signaling and attenuate integrin α_IIb_β_3_ outside–in signaling functions with no consequence on hemostasis and thrombosis.

Loss of PP1cα modestly reduced activation of integrin, binding of soluble fibrinogen, and aggregation of platelets especially to a low dose of platelet agonist (Figure 1, Figure 2 and Figure 3). These findings are consistent with our previous report of reduced binding of soluble fibrinogen and aggregation of platelets by PP1cα^−/−^ platelets to low-dose thrombin (0.02 U/mL) [16]. It remains to be understood whether higher agonist concentrations initiate multiple redundant signaling pathways in PP1cα^−/−^ platelets and trigger activation of PP1cβ or PP1cγ isoforms to assist platelet activation. Indeed, in our previous study with PP1cγ^−/−^ mice model, we also observed reduced inside–out signaling selectively to a low dose of thrombin [19]. Thus, unlike the robust inhibition of platelet inside–out signaling functions observed in human platelets with pharmacological agents, genetic loss of PP1cα in murine platelets only displayed a subtle reduction in inside–out signaling.

In contrast, loss of PP1cα in murine platelets moderately enhanced integrin α_IIb_β_3_ outside–in signaling functions (Figure 4 and Figure 5). Interestingly, these platelet studies validate our previous observations in 293 α_IIb_β_3_ overexpressing heterologous cells, wherein selective depletion of PP1cα caused increased outside–in signaling functional effects (adhesion and clot retraction) [23]. Enhanced outside–in signaling phenotype in PP1cα^−/−^ murine platelets indicates that this phenotype is intrinsic to PP1cα and independent of the cell model we previously employed. Importantly, unlike the inhibition of outside–in signaling functions in human platelets treated with inhibitors of Ser/Thr phosphatases, genetic loss of only PP1cα moderately potentiated integrin α_IIb_β_3_ mediated outside–in signaling function.

How PP1cα can amplify inside–out signaling and attenuate outside–in signals remains unclear. PP1cα spatially and temporally regulate platelet inside–out and outside–in signaling pathways in part by engaging with phosphatase interacting proteins (PIP) and subsequent phospho-modulation of specific effectors within the pathway. Indeed, we previously demonstrated the Gβ_1_ protein as one PIP downstream of the G protein-coupled receptor. Thrombin stimulation led to the dissociation of PP1cα from Gβ_1_, with subsequent association and dephosphorylation of phospholipase C β_3_ (PLCβ_3_) at Ser 1105. Since Ser 1105 phosphorylation blocks signaling, dephosphorylation by PP1cα may amplify agonist signaling [16]. In the context of outside–in signaling pathway, integrin α_IIb_ [25], cytoskeletal protein tensin 1 [26] and p38 MAPK [23] represents some of the PIP. Consistent with the contribution of p38 MAPK in outside–in signaling [27] we observed increased p38 MAPK activation in fibrinogen-adhered PP1cα^−/−^ platelets (Figure 4) and p38 inhibitor to block the enhanced outside-on signaling functions (Figure 5). It is possible that loss of PP1cα may impact tensin 1-induced cytoskeletal reorganization in platelets to potentiate outside–in signaling, as there is precedence for tensin 1 to engage integrin β3 [28] and disruption of PP1cα-tensin interaction enhanced cell migration and invasion of cancer cells [29]. It is important to emphasize here that the distinct phenotype displayed by the loss of PP1cα is not a reflection of perturbation by one effector in a particular pathway. In fact, PP1c interacts with more than 200 phosphatase-interacting proteins (PIP) in a cell. Several of the PIPs represent endogenous PP1 inhibitors, which can be regulated by phosphorylation events [30]. Given the complexity of the PP1c interactome and its ability to integrate with other signaling networks, the functional outcome of PP1cα loss is likely to be more than an additive effect displayed by the phospho-modulation of several PP1cα effectors along the pathway.

Despite the critical role of platelet activation in hemostasis and thrombosis, in vivo hemostasis and thrombosis were unaffected in PP1cα^−/−^ mice (Figure 7). Opposing roles for PP1cα in two signaling pathways that contribute to hemostasis and thrombosis along with potential compensation by PP1cβ and/or PP1cγ isoforms in platelets may have contributed to the lack of an in vivo effect. Supporting the issue of compensation by other PP1c isoforms, PP1cγ^−/−^ global mice had no defect in in vivo hemostasis and only moderately reduced time to occlusion in a light-dye injury model [19]. Despite no differences in the occlusion time in vivo, ex vivo coverage of whole blood on immobilized collagen was enhanced in PP1cα^−/−^ mice. This suggests that platelet PP1cα can crosstalk with injured endothelial cells during the development of thrombosis in vivo and modulate outcome.

## 5. Conclusions

Despite the subtle phenotype, our studies suggest that PP1cα contributes to platelet signaling in a fashion that is specific to the mode of signaling. Specifically, PP1cα supports agonist induced inside–out signaling and opposes integrin-mediated outside–in signaling. Interestingly, recent studies suggest a role for PP1 signaling in platelet–cancer cross-talk [31], and alterations in platelet transcriptome including the regulatory subunits of PP1 occur during sepsis [32]. Thus, a contrasting and pleiotropic effect of platelet PP1cα may have relevance to platelet functions beyond hemostasis and thrombosis.

## Data Availability

All data and materials will be available upon request.
